# Identification of Immune-Related Genes and Development of SSR/SNP Markers from the Spleen Transcriptome of *Schizothorax prenanti*

**DOI:** 10.1371/journal.pone.0152572

**Published:** 2016-03-28

**Authors:** Hui Luo, Shijun Xiao, Hua Ye, Zhengshi Zhang, Changhuan Lv, Shuming Zheng, Zhiyong Wang, Xiaoqing Wang

**Affiliations:** 1 College of Animal Science & Technology, Hunan Agricultural University, Changsha, Hunan, China; 2 Fisheries Breeding and Healthy Cultivation Research Centre, Southwest University, Chongqing, China; 3 Key Laboratory of Healthy Mariculture for the East China Sea, Ministry of Agriculture, P.R. China, Fisheries College, Jimei University, Xiamen, Fujian, China; 4 Collaborative Innovation Center for Efficient and Health Production of Fisheries in Hunan Province, Changde, Hunan, China; Macquarie University, AUSTRALIA

## Abstract

*Schizothorax prenanti* (*S*. *prenanti*) is mainly distributed in the upstream regions of the Yangtze River and its tributaries in China. This species is indigenous and commercially important. However, in recent years, wild populations and aquacultures have faced the serious challenges of germplasm variation loss and an increased susceptibility to a range of pathogens. Currently, the genetics and immune mechanisms of *S*. *prenanti* are unknown, partly due to a lack of genome and transcriptome information. Here, we sought to identify genes related to immune functions and to identify molecular markers to study the function of these genes and for trait mapping. To this end, the transcriptome from spleen tissues of *S*. *prenanti* was analyzed and sequenced. Using paired-end reads from the Illumina Hiseq2500 platform, 48,517 transcripts were isolated from the spleen transcriptome. These transcripts could be clustered into 37,785 unigenes with an N50 length of 2,539 bp. The majority of the unigenes (35,653, 94.4%) were successfully annotated using non-redundant nucleotide sequence analysis (nt), and the non-redundant protein (nr), Swiss-Prot, Gene Ontology (GO), and Kyoto Encyclopedia of Genes and Genomes (KEGG) databases. KEGG pathway assignment identified more than 500 immune-related genes. Furthermore, 7,545 putative simple sequence repeats (SSRs), 857,535 single nucleotide polymorphisms (SNPs), and 53,481 insertion/deletion (InDels) were detected from the transcriptome. This is the first reported high-throughput transcriptome analysis of *S*. *prenanti*, and it provides valuable genetic resources for the investigation of immune mechanisms, conservation of germplasm, and molecular marker-assisted breeding of *S*. *prenanti*.

## Introduction

Fish of the subfamily Schizothoracinae (Teleostei: Cyprinidae) are mainly distributed in rivers on the Qinghai-Tibetan Plateau and its peripheral areas [1] in China. These fish are well adapted to the harsh conditions of the Plateau [2] and have evolved many specific traits to adapt to an environment that exposes them to low temperatures, high levels of radiation, and hypoxia [1]. As a consequence, Schizothoracinae fish are regarded as excellent models to study high altitude adaptations in animals [3]. *Schizothorax prenanti* is a member of the subfamily Schizothoracinae and is mainly distributed in the upstream regions of the Yangtze River and its tributaries in China. The species lives in a cold-water environment with a gravel riverbed [4]. Given the extreme environmental changes in its habitat areas, *S*. *prenanti* offers an excellent model to investigate the effects of historical and contemporary environmental changes [5]. *S*. *prenanti* is also important commercially in west China because of its high flesh quality and good flavor. In recent years, it has become one of the most important cold-water aquaculture species in China. However, overly dense stocking levels and rapid expansion of aquaculture has led to several problems affecting the sustainable development of the industry, such as the frequent outbreak of infectious diseases [6,7]. Concurrently, the wild resources and populations of *S*. *prenanti* have rapidly declined because of water pollution and the construction of hydropower stations [8–10]. In order to reduce economic losses caused by infective agents and to protect germplasm resources, it will be necessary to identify genes that have a role in economically important traits, including the immune system. The development of molecular markers for use in selective breeding programs will also be of importance.

Transcriptome sequences can be used to identify genes and to develop genetic molecular markers [11]. The recent advances in next generation sequencing (NGS) technologies have enabled the transcriptomes of non-model species to be analyzed in a high-throughput manner. The ability to sequence all transcripts in one experiment and to assess gene expression levels has led to the application of high-throughput methods to species of importance for aquaculture [12–15]. To date, RNA sequencing (RNA-Seq) has been employed in a wide range of aquatic animals [11,16] to examine immune responses [17–19], growth and development [20–23], evolution [3,24,25], and toxicology [26,27]. Taxonomy, diversity, geographic distribution, and disease prevention have been examined in *S*. *prenanti* [28]. In recent years, microsatellite markers have been developed from a small number of expressed sequence tags (ESTs), and several genes involved in growth, metabolism, and immunity have been characterized [5,28–33]. However, a detailed transcriptome analysis has still not been undertaken in this important fish species. The identification of genes involved in the immune system and the development of molecular markers will significantly advance investigations into the *S*. *prenanti* immune defense mechanisms and also contribute to selective breeding programs for this species.

In this study, we used the Illumina Hiseq2500 platform to analyze the spleen transcriptome of *S*. *prenanti*. Genes involved in immune pathways were identified; the majority of these are reported for the first time. In addition, we identified and analyzed SSR markers and small variants (SNPs/InDels) in the transcriptome. This is the first analysis of a transcriptome from *S*. *prenanti* and will enable large-scale molecular marker development.

## Materials and Methods

### Fish and sample collection

Eighteen one-year-old *S*. *prenanti* individuals (average body weight 105 g, average body length 18.7 cm) were collected from an aquaculture farm in Meishan, Sichuan Province, China. The fish were reared in a recirculating freshwater system (18 individuals/tank; tank dimensions 100 × 48 × 60 cm) at the Fisheries Breeding and Healthy Cultivation Research Centre of Southwest University. The fish were maintained at 19 ± 1°C in aerated water for two weeks before the experiment. They were fed a commercial diet (Sichuan Giastar Group; particle diameter 2 mm) twice a day. The experimental protocols used here were approved by the institutional animal care and use committee of Southwest University. In order to reduce stress, fish were anesthetized using tricaine methanesulfonate (MS222) before dissection. Spleen tissues were randomly mixed into three samples (6 individuals for each sample) and stored in 1 mL Sample Protector for RNA (TaKaRa, Dalian, China) at 4°C overnight. The samples were then transferred to a -80°C ultra-low freezer until preparation of RNA.

### RNA extraction, cDNA library construction, and Illumina sequencing

Total RNA was extracted using TRIzol reagent (Invitrogen, USA) and incubated for 1 h at 37°C with 10 units of DNase I (TaKaRa, Dalian, China) to eliminate genomic DNA. RNA quality and quantity were analyzed using a BioAnalyzer 2100 (Agilent Technology, Santa Clara, CA) and NanoDrop 2000 spectrophotometer (Infinigen Biotechnology Inc., City of Industry, CA), respectively. In order to evaluate the reliability of the libraries, we constructed three cDNA libraries using spleen tissue from six fish per RNA library. Poly(A)^+^ RNA was purified with oligo(dT) magnetic beads and fragmented into short sequences. First-strand cDNA was synthesized using random hexamer primers and Superscript III (Invitrogen, Carlsbad, CA, USA); this was followed by second-strand cDNA synthesis, end repair, and adaptor ligation. Finally, libraries with insert lengths of ~280 bp were created by PCR amplification and purification. Each library was sequenced on an Illumina Hiseq2500 in 125PE mode (Illumina Inc., San Diego, CA, USA). Short reads were deposited in the NCBI Sequence Read Archive (SRA) under Accession numbers SRR2241952, SRR2241953, and SRR2241954.

### Transcriptome de novo assembly and annotation

In order to ensure reliable assembly results, the quality of raw reads was checked by FastQC (http://www.bioinformatics.babraham.ac.uk/projects/fastqc/) and filtered by the high-throughput quality control (HTQC) toolkit [34]. The following quality-filtering criteria were applied: 1) the 5-bp window was omitted if the average quality was lower than 20; 2) reads were removed if the percentage of unknown bases was higher than 10%; 3) read pairs were filtered if any one read end was shorter than 50 bp. The resulting cleaned reads were used in the following bioinformatics pipelines. The Trinity package was used for transcript assembly using default parameters [35]. The assembled transcripts were then processed through the Evigene package (http://arthropods.eugenes.org/EvidentialGene/) to eliminate sequence redundancy with default parameters [36]. The resulting transcripts that showed significant similarities (>90%) were then clustered and the longest transcripts for each group were selected as representative unigenes, which were then used for functional annotation. Sequence-length statistics of the assembled transcriptome were performed using our own Python scripts. The assembled sequences have been submitted as S1 Text. For gene annotation, BLAST package (with an E-value threshold of 1×10^−5^) of all unigenes were performed in the National Center for Biotechnology Information (NCBI) non-redundant nucleotide sequence (nt) and non-redundant protein (nr) databases [37]. Transcripts were further annotated using the Swiss-Prot, Gene Ontology (GO), EC (Enzyme Code), and Kyoto Encyclopedia of Genes and Genomes (KEGG) databases with Blast2GO [38]. The gene annotations in NCBI Nr/Nt and Swiss-prot were available in S2 Text.

### SSR, SNP, and InDel discovery

To develop SSR markers from the transcriptome of *S*. *prenanti*, sequences with repeat unit lengths from di- to hexa-nucleotides were detected using the MicroSAtellite application (MISA, http://pgrc.ipk-gatersleben.de/misa/). The parameters were set to identify di-, tri-, tetra-, penta-, and hexa-nucleotide motifs with a minimum of six, five, five, five, and five repeats, respectively. To eliminate non-genomic artificial SSR loci caused by the sequence complexity of the transcripts, mono-nucleotide SSR markers were excluded in this work. For the development of putative SNPs and InDels, BWA 0.7.6a [39], SAMTools 1.19 [40] and GATK 2.8–1 [41] pipeline methods were applied. Only SNP and InDel markers with a depth greater than 5 and a quality score higher than 100 were selected as reliable loci for subsequent analysis.

### Immune gene PCR validation

To validate the reliability of the assembly of the transcriptome, 15 annotated unigenes related to immunity were selected and validated using PCR experiments. Primer set was designed based on RNA-Seq unigenes sequences by Primer Premier 5.0. Total RNA was extracted from spleen tissues of 18 fish (3 biological replicate sample pools (n = 6 fish for each pools)). First strand cDNA was synthesized from 4 μg total RNA and used as a template for PCR with gene-specific primers. The PCR analysis was performed on the Applied Biosystems 2720 Thermal Cycler using Ex Takara DNA Polymerase according to the manufactureʹs protocol. PCR amplifications were performed in 10 μL reactions, containing genomic DNA 1.0 μL, 10× PCR buffer 1.0 μL, 15 mM MgCl_2_ 1.0 μL, 0.2 μM dNTPs, 0.2 μM of each primer, 5 U Taq enzymes 0.1μL and ddH_2_O 7.3 μL. The PCR reaction procedure was that 94°C for 5 min, 30 cycles at 94°C for 0.5 min, annealing temperature (from 55°C to 62°C) for 0.5 min, 72°C for 0.5 min and a final extension step at 72°C for 10 min.

## Results and Discussion

### Transcriptome sequencing and assembly

The three cDNA libraries yielded a total of 99.6 million raw reads with a read length of 125 bp, resulting in a total of 12.5 Gb. Sequencing quality was assessed using FastQC (http://www.bioinformatics.babraham.ac.uk/projects/fastqc/) to determine the Phred quality score of each base in the raw reads. More than 93% of the bases had a Phred quality greater than 20, with 88% having a score greater than 30 (S1 Fig). After read quality evaluation and length trimming (see Method and Material for detail), 97.3 Mb cleaned pair-ended reads were obtained and used for the bioinformatics analysis.

**Fig 1:**
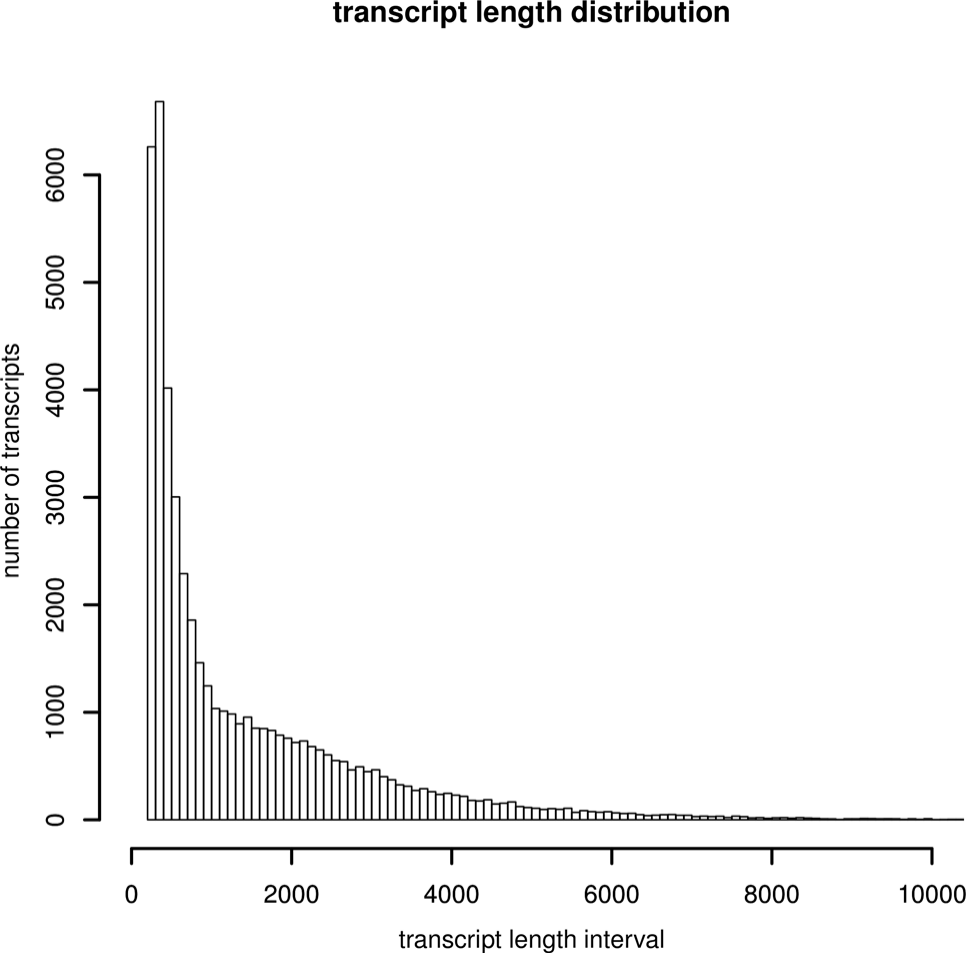
Length distribution of assembled transcripts. Assembled transcript numbers (Y-axis) were plot against length interval (X-axis).

The Trinity package [35] was used for *de novo* transcriptome assembly from the cleaned reads. An additional step with Evigene [36] was used to remove redundancy in the assembled contigs. As a result, we obtained 48,517 transcripts ranging from 201 to 27,365 bp with an average length of 1,491 bp (Table 1, Fig 1). The transcripts clustered into 37,785 unigenes with an average length of 1,323 bp (ranging from 201 bp to 27,365 bp) (Table 1). The N50 lengths of transcripts and unigenes were 2,700 and 2,539 bp, respectively; these estimates are comparable with those reported previously for other fish species [11,42]. Approximately 50% of transcripts ranged from 201 to 500 bp (Fig 1). An estimated 21,704 (44.7%) transcripts exceeded 1,000 bp and 18,199 (26.3%) exceeded 2,000 bp; these proportions are consistent with a previous study [43].

**Table 1:** Assembled transcripts and unigenes obtained from transcriptome analysis.

Terms	Transcripts	Unigenes
Total number	48,517	37,785
Shortest length (bp)	201	201
Longest length (bp)	27,365	27,365
N50 length (bp)	2,700	2,539

**Fig 2:**
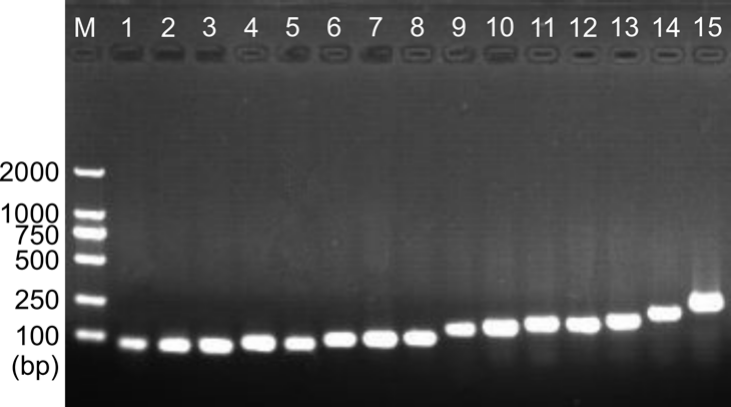
PCR amplification and agarose gel (1%) electrophoresis of 15 unigenes. The corresponding run lanes of unigene 8059, unigene 11048, unigene 25066, unigene 24999, unigene 10116, unigene 24705, unigene 12568, unigene 19017, unigene 16797, unigene 22539, unigene 17508, unigene 11117, unigene 24689, unigene 17711 and unigene 23056 are from 1 to 15, respectively.

To validate the reliability of the assembly of the transcriptome, 15 annotated unigenes related to immunity were selected to be analyzed using PCR experiments. Primers were designed based on corresponding unigenes sequences, their putative gene names, primer sequences and expected PCR product sizes are shown in Table 2. As we expected, PCR experiments exhibited amplicons of expected sizes for all 15 unigenes (Fig 2). The results not only testified the reliability of the assembly of the transcriptome and transcript annotation, but also indicated that it could be useful for further research.

**Table 2:** Putative gene name and primer sequences and the expected size for PCR of the 15 unigenes.

Number	Putative gene name	Forward primer	Reverse primer	Product size (bp)
unigene8059	Interleukin 12A	TGCTGGAGATGGACCACTTG	ACACCGTTTGCGTCCTGAT	81
unigene11048	Complement C1q subcomponent subunit A	TTTTCTTTGGGTGCTGCTTC	GACTCCATTCACGCCATCTTT	82
unigene25066	Toll-like receptor 5	TGATATTAAGTGGGTAGAAAGAGCG	CAAAGCAGCAGCGGAGTGT	83
unigene24999	Major histocompatibility complex, class I	CCCATCCTAAACCAGTACACCA	GCCTTTTCGGACCTCCTCAT	99
unigene10116	C-C motif chemokine 21	ATGGTTTACTGGTGTCTATGCTTCT	CCAAGTGACAATAACTGGGTGAG	101
unigene24705	Interleukin 6 receptor	CCACCTGTAACCATAAGAAAAGAAC	TTGCTCAAAATCTGTCCCCAT	120
unigene12568	Myeloid differentiation primary response protein MyD88	AGACTGAACGCAACCTGAAGC	GAAATGACCACCACCATCCTC	121
unigene19017	Interleukin 1 receptor type II	TACAGGAGATTGCGAGTGAACA	AGATGGGTGAGACGGGAGGA	126
unigene16797	Heat shock 70kDa protein 1/8	TGGAGGGAAGCCGAAAG	TCATTGAAATAGGCAGGAACTG	166
unigene_22539	Toll-like receptor 4	AACAACATGCCCATGACCTTT	GCTTATCCGCCATCCCTATCT	175
unigene17508	Tumor necrosis factor, alpha-induced protein 3	ACAAATCAAACCTCGTCCTCG	CACAGCCCGTCATACTCCAC	182
unigene11117	Toll-interacting protein	ATCCAGAGCACTGTCCCACC	TGCCTTCTTTGTCATCACCCT	184
unigene24689	Integrin alpha 5	GGAGTTGGGTCGGGTCTATG	TGAGCACCAGTCCTTGTTTGTT	200
unigene17711	TNF receptor-associated factor 6	CTCAGTTGCCGCCCATTCT	TCGGGTGTCCTCCTCCAAA	232
unigene23056	Toll-like receptor 3	GCAGTTCCAACGGACTTACCC	GCCCATTTAGCTTCAGCCTATT	283

### Transcript annotation

**Fig 3:**
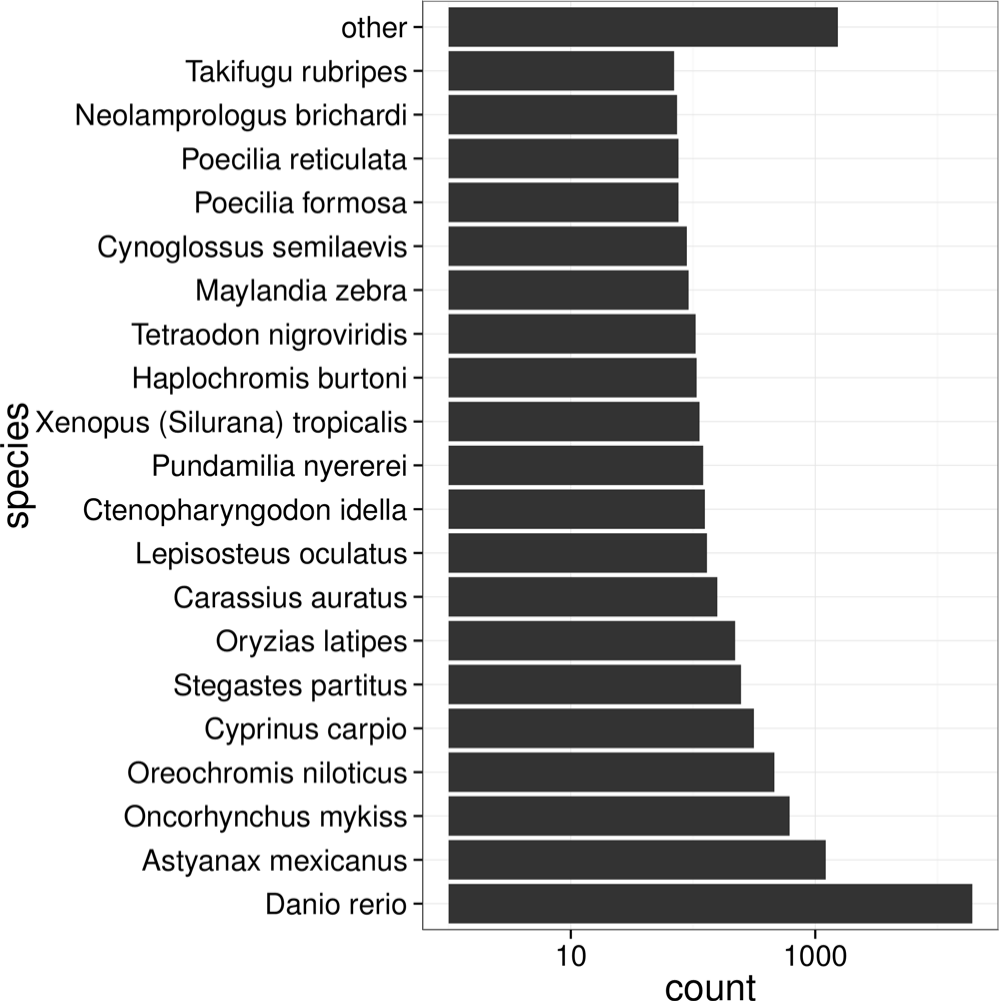
Species distribution for NCBI nr databases annotation. Note that only the best hits for unigenes were used in the analysis.

Functional annotation of the transcriptome was carried out by searching the transcripts against nucleotide and protein databases. As a result, 34,813 (92.1%), 25,222 (66.7%), and 20,765 (54.9%) unigenes showed significant similarities (E value_1× 10^−5^) to the NCBI nucleotide (nt), protein (nr) and Swiss-Prot databases, respectively (Table 3). We found that the majority (19,272, ~76%) of homology hits in the nr search were to zebrafish (*Danio rerio*), a result that is consistent with the fact that both *S*. *prenanti* and zebrafish belong to the Cyprinidae family and therefore have a close evolutionary relationship. In addition, 4.8% (1,219) of hits in the nr database were from *Astyanax mexicanus*, 2.4% (617) from *Oncorhynchus mykiss*, 1.8% (463) from *Oreochromis niloticus*, and 1.2% (315) from *Cyprinus carpio* (Fig 3).

**Table 3:** Unigenes annotation by various databases.

Database	Hit number	Percentage (%)
Nr	25,222	66.7
Nt	34,813	92.1
Swiss-prot	20,765	54.9
GO	21,760	57.6
KEGG	5,445	14.4
EC	2,648	7.1
Total	35,653	94.4

**Fig 4:**
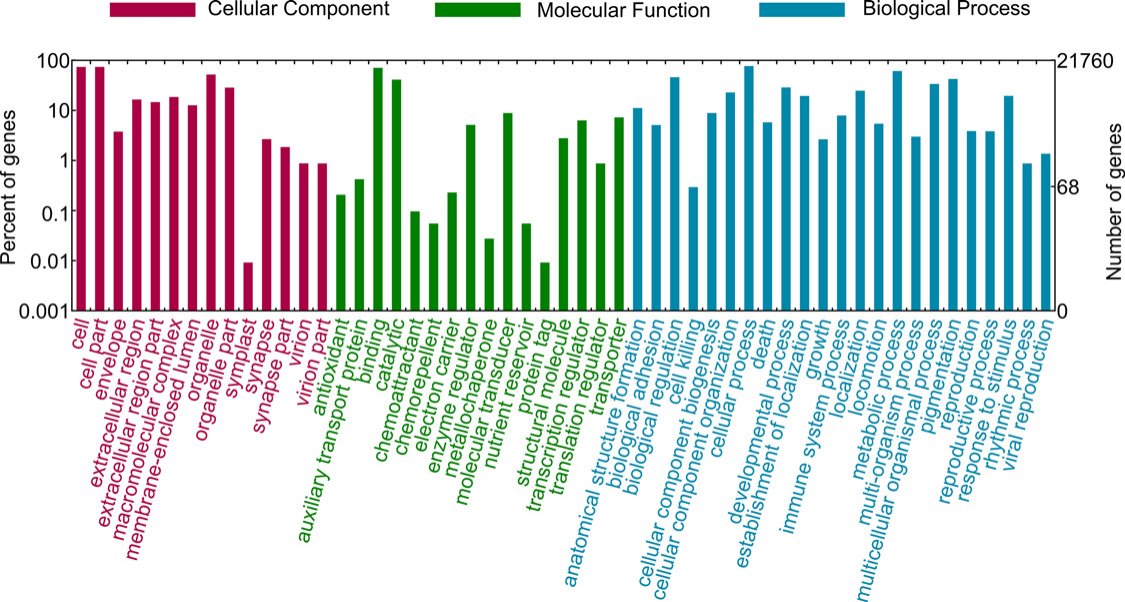
GO annotation of prenant’s schizothoracin transcriptome. Unigenes were annotated by Gene Ontology (GO) terms which belong to three main categories: biological process, cellular component, or molecular function.

The potential functions of the unigenes were determined using Gene Ontology (GO) databases. GO classification generally assigns the functions of genes and their products in organisms. The spleen transcriptome unigenes were annotated to three major GO categories—16,982 unigenes (44.9%) were assigned to Cell Component (CC), 19,229 (50.9%) to Molecular Function (MF), and 18,948 unigenes (50.1%) to Biological Process (BP) (Table 3; Fig 4). The most enriched components in CC terms were cell (16,101 unigenes, GO: 0005623), cell part (16,100 unigenes, GO: 0044464), and organelle (11,324 unigenes, GO: 0043226). For MF terms, a large number of unigenes were assigned to binding (15,426 unigenes, GO: 0005488), catalytic activity (8,963 unigenes, GO: 0003824), and molecular transducer (1,930 unigenes, GO: 0060089). In the BP category, most of the unigenes were related to the terms cellular process (16,734 unigenes, GO: 0009987), metabolic process (13,315 unigenes, GO: 0008152), and biological regulation (10,027 unigenes, GO: 0065007) (Fig 4). These results indicated that the annotated unigenes were assigned to various terms of the biological process category as has been reported in previous transcriptome analyses of the larger yellow croaker (*Larimichthys crocea*), the naked carp (*Gymnocypris przewalskii*), and the blunt snout bream (*Megalobrama amblycephala*) [11,42,44].

**Fig 5:**
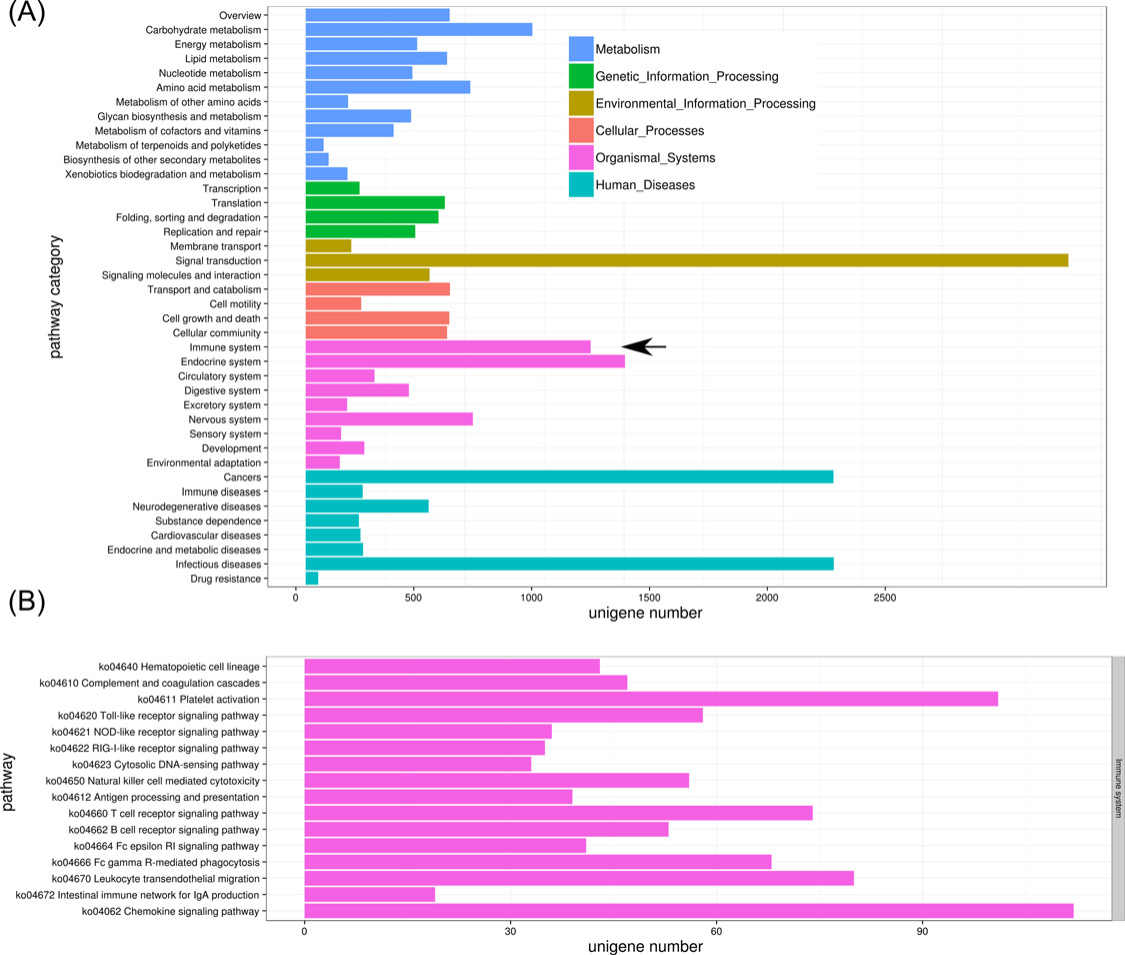
Identified KEGG pathways of assembled unigenes. Unigene numbers distribution in six major categories: Metabolism, Genetic Information Processing, Environmental Information Processing, Cellular Processes, Organismal Systems, Human Diseases. Immune system was indicated by black arrow(A); the detailed unigenes distribution in 16 immune-related pathways (B).

Next, the unigenes were mapped to reference canonical pathways in the KEGG database; 5,445 (14.4%) unigenes were assigned to KEGG Orthology (KO) terms and grouped into 365 pathways. The annotated pathways were clustered into six major categories: Metabolism, Genetic Information Processing, Environmental Information Processing, Cellular Processes, Organismal Systems, and Human Diseases. The detailed pathways and distributions in each major pathway category are shown in Fig 5A. KEGG pathway-based analysis facilitates the systematic study of intricate metabolic pathways and the biological behavior of functional molecules. The largest pathway in the present annotation was ‘Pathways in cancer’ (ko05200), which contained 269 unigenes. Other major pathways were ‘PI3K-Akt_signaling_pathway’ (218 unigenes, ko04151), ‘Purine metabolism’ (200 unigenes, ko00230), ‘HTLV-I infection’ (193 unigenes, ko05166), and ‘Biosynthesis of amino acids’ (183 unigenes, ko01230). These results are similar to those reported for the Eastern Oyster (*Crassostrea virginica*) [18]. In addition, Enzyme Commission (ECs) were assigned to 2,648 (7.1%) unigenes according to KEGG mapping results.

### Identification of immune-related genes

KEGG pathway assignments were used to identify functional unigenes involved in immune processes and their interactions. In total, 511 immune-related unigenes were identified in 16 KEGG immune pathways (Fig 5B). Many of these unigenes are reported for the first time in *S*. *prenanti*. The number of immune genes identified here is similar to that reported in the Miiuy Croaker (*Miichthys miiuy*) [45] and rainbow trout (*Oncorhynchus mykiss*) [46]. Two functional subcategories of immune response, ‘Chemokine signaling pathway’ (112 unigenes, ko04062) and ‘Platelet activation’ (101 unigenes, ko04611), had most unigenes. Other important immune pathways with large numbers of unigenes included ‘Toll-like receptor signaling pathway’ (58 unigenes, ko04620), ‘Complement and coagulation cascades’ (47 unigenes, ko04610), ‘Leukocyte transendothelial migration’ (80 unigenes, ko04670), ‘T-cell receptor signaling pathway’ (74 unigenes, ko04660), ‘B-cell receptor signaling pathways’ (53 unigenes, ko04662), and ‘Fc gamma R-mediated phagocytosis’ (68 unigenes, ko04666). The detailed pathways, KO terms, putative functions, and expression of these immune-related genes are summarized in S1 Table. To systematically identify genes involved in these pathways, we identified genes in several representative networks, including Toll-like receptor signaling pathway, Complement and coagulation cascades, and Chemokine signaling pathway.

### Toll-like receptor signaling pathway

Toll-like receptors (TLRs) are a type of pattern recognition receptor (PRR) and are key components of the innate immune system. They were the first PRRs to be characterized [47]. TLRs detect the presence of pathogens through recognition of pathogen associated molecular patterns (PAMPs) and trigger innate immune responses [46,48]. To date, 17 TLRs have been identified in teleost species [49]. Bacterial PAMPs are mainly detected by TLR1, TLR2, TLR4, TLR5, TLR6, TLR7, and TLR9 [50] and nucleic acids are recognized by TLR7/8, TLR3, and TLR9 [51]. Here, we identified five unigenes showing high similarity to TLR3, TLR4, TLR5, TLR7, and TLR8. Notably, TLR4 has been reported only in zebrafish and the Chinese rare minnow (*Gobiocypris rarus*) and not in other teleosts [49,52]. Our results suggest that *S*. *prenanti* is likely to be a teleost species that possesses TLR4, similar to zebrafish and Chinese rare minnow. TLR4 plays an important role in functional links between TLRs and complement in mammalian immune systems. In a tissue-damage model of the mouse intestine, it was observed that TLR4 stimulation could modulate local C3 and factor B synthesis [53]. TLR4-mediated signaling is also involved in T-helper 17 cell differentiation [54]; however TLR4 has rarely been reported in fish and the cross-talk between complement and TLRs has not yet been described in teleosts. We found that *S*. *prenanti* had a TLR4 homologous to that in zebrafish; therefore, investigation of the teleost-specific relationship between complement and TLRs will be an interesting topic in fish immunological studies. The gene identification from the *S*. *prenanti* transcriptome may not represent the complete set of TLRs in this species and further studies are needed to identify more TLR genes for a better understanding of the molecular immune mechanism. All unigenes involved in the Toll-like receptor signaling pathway are listed in S2 Fig and S1 Table.

### Complement and coagulation cascades

In general, the complement system is considered the first line of defense against microbial invaders [55]. The complement system includes over 20 soluble and cell-surface proteins that respond to the presence of foreign antigens by activating a regulated cascade of reactions [56]. There are three different ways to initiate the complement system on the surface of invading pathogens—the classical, alternative, and mannan-binding lectin pathways. Although activation of each pathway depends on different factors, they all produce the same anti-infection effects [57]. In this study, three unigenes were found to have high similarity to C3, C4, and C5. Complement components C3, C4, and C5 belong to the alpha-2 macroglobulin superfamily of thioester-containing proteins [58]. C3 is the key element of the complement system and when activated can split into C3a and C3b in the three different pathways [59]. C4 serves as a link in the initiation of the lectin and classical pathways [56]. C5 plays a pivotal role in the formation of the membrane attack complex (MAC), which results in cell lysis [59,60]. In addition to the above complement members, we also found many other key genes in complement and coagulation, such as C6, C7, C8, C9, complement C1q subcomponent subunit A (C1QA), complement C1q subcomponent subunit C (C1QG), Factor B, and Factor D. However, excessive complement activation can cause serious damage to the host tissue, resulting in anaphylaxis and cell damage. Therefore, complement activation needs to be controlled in multiple reaction steps by several different regulatory elements. For instance, C1-inhibitor (C1INH), factor H, membrane-cofactor protein (MCP), C4-binding protein (C4bp), S-protein, and CD59 are key regulatory elements [55]. In this study, we identified several regulatory factors: C1-inhibitor (C1INH), S-protein, and CD59. Information on unigenes involved in Complement and coagulation cascades is included in S3 Fig and S1 Table.

### Chemokine signaling pathway

Chemokines are chemotactic cytokine family components that are secreted by tissues at an early stage of infection. These secreted chemokines are small heparin-binding molecules that recruit neutrophils, monocytes, and other effector cells from vessels towards the focus of infection [57]. The two most important and studied families of chemokines are CC chemokines (characterized by two adjacent cysteine residues next to the N-terminus) and CXC chemokines (characterized by two cysteine residues separated by one amino acid next to the N-terminus). These chemokine families and their receptors have been found in many bony fish species [57]. Here, we found seven, five, one, and four unigenes showing high similarity to CC, CC chemokine receptors (CCR), CXC, and CXC chemokine receptors (CXCR), respectively. The number of predicted CC chemokines from *S*. *prenanti* was significantly less than that (26) in catfish [61], suggesting that further transcriptome analysis under infectious conditions will be needed to identify further genes in the Chemokine signaling pathway. Several CXC chemokines, including CXC12 and CXC14, have been identified in fish [62,63]; however, only one *S*. *prenanti* unigene showed high similarity to CXC11 and none were homologous to CXC12 or CXC14. In addition, we identified three CXCRs (CXCR3, CXCR4, and CXCR5) in *S*. *prenanti*. Unigenes involved in the chemokine signaling pathway are listed in S4 Fig and S1 Table.

Although some representative immune genes, such as myeloid differentiation factor 88 (MYD88) gene [33], were identified here, most of the immune-related genes found in this study have not previously been reported in *S*. *prenanti*. These immune-related genes offer a valuable resource for further gene function investigations to explore the detailed immune mechanism in this species. We also noted that some important immune genes identified in other fish were absent in our transcriptome; the absence of these genes may indicate that they are species-specific; alternatively, since all the sampled fish in this study were healthy, then genes with a zero or low expression level would not be included in the RNA-Seq and gene assembly. It will be necessary to repeat this analysis under different physiological conditions to identify further genes and to examine their expression patterns.

### SSR, SNP, and InDel discovery

SSRs are useful molecular markers for genetic and breeding studies. The development of genetic markers is the first step in the application of genomic resources to improve a broodstock [64]. At the present time, only a few SSR markers are available for *S*. *prenanti* [5,30]. Here, we detected potential SSR markers using the MISA package (http://pgrc.ipk-gatersleben.de/misa/). As it was difficult to distinguish true mononucleotide repeats from polyadenylation sites and false-positive mononucleotide repeats generated by sequencing errors, we did not include mononucleotide repeats in the following analysis.

**Fig 6:**
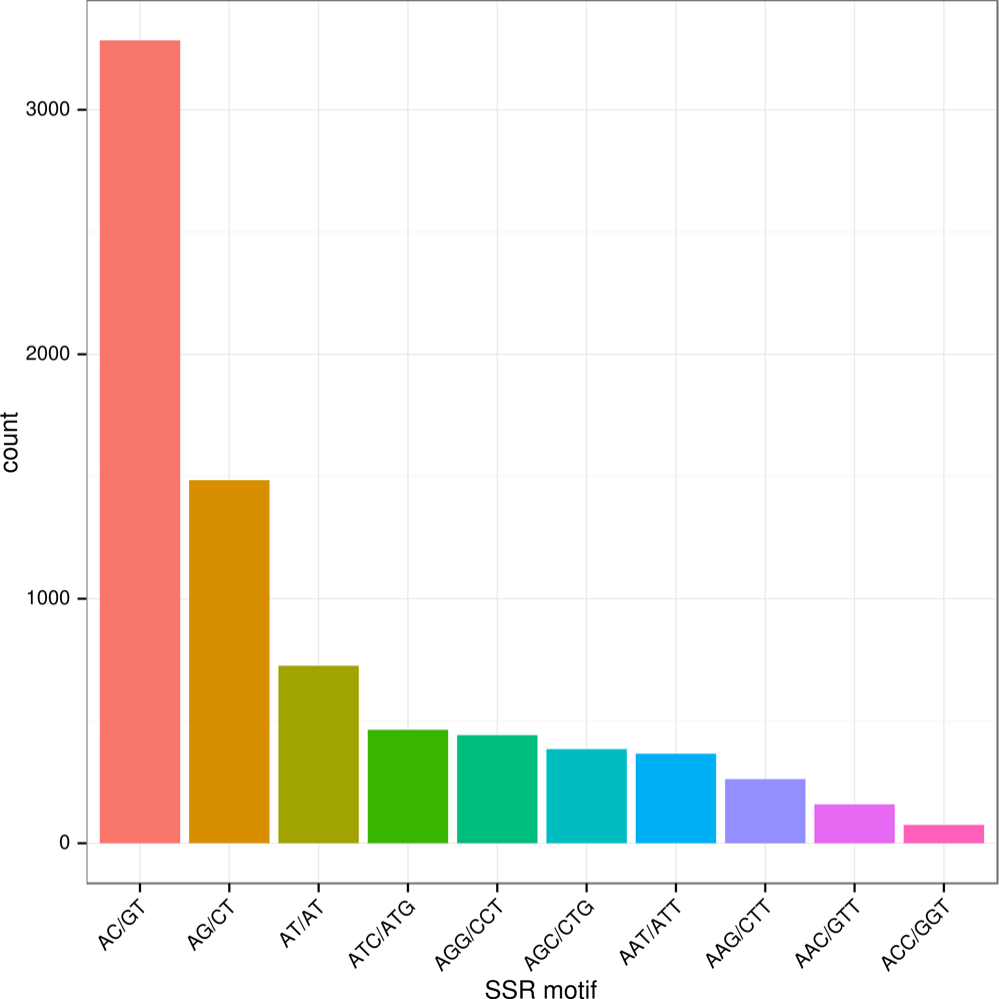
Frequency distribution of the top ten most abundant SSRs based on motif sequence types. Each histogram represented one detected SSR type in transcriptome of *S*. *prenanti*. Sequence complementary was considered during SSR type classification.

A total of 7,545 SSRs of 2–6 bp unit length were identified (Table 4); this number of SSRs corresponds to a frequency of about one SSR per 9.6 kb of expressed sequences. We identified 5,168 (68.5%) di-nucleotide repeats, 2,131 (28.2%) tri-nucleotide repeats, and 246 (3.2%) tetra-/penta-/hexa-nucleotide repeats. The AC/GT sequence was the most common among the di-nucleotide repeats motifs, followed by AG/CT and AT/AT (Fig 6). Ten types of tri-nucleotide repeats were found; ATC/ATG was the most abundant, followed by AGG/CCT and AAT/ATT (Fig 6 and S2 Table). The complete set of SSR units and repeat number distributions is listed in S2 Table. The pattern of SSRs found here is consistent with that reported in previous studies in blunt snout bream (*Megalobrama amblycephala*) [64], but differs from those in yellow catfish (*Pelteobagrus fulvidraco*) [65] and half-smooth tongue sole (*Cynoglossus semilaevis*) [66], indicating that SSR repeat unit distributions are likely to be species-specific in teleosts. As is shown in Table 4, the majority of SSRs (69.27%) have a repeat number lower than 8.

**Table 4:** Repeat numbers and unit length distribution of putative SSR markers in the transcriptome.

Repeat numbers	Motif length					Total	Percent %
	Di	Tri	Tetra	Penta	Hexa		
5	-	1,368	227	6	5	1,606	20.08
6	1,999	507	22	1	1	2,530	31.63
7	1,090	314	-	-	-	1,404	17.55
8	867	15	-	-	1	883	11.04
9	891	1	-	-	-	892	11.15
10	535	2	-	-	1	538	6.73
11	137	-	-	-	-	137	1.71
≥12	3	4	1	-	-	8	0.10
Total	5,522	2,211	250	7	8	7,998	100
Percent %	69.04	27.64	3.13	0.09	0.10	100	

**Fig 7:**
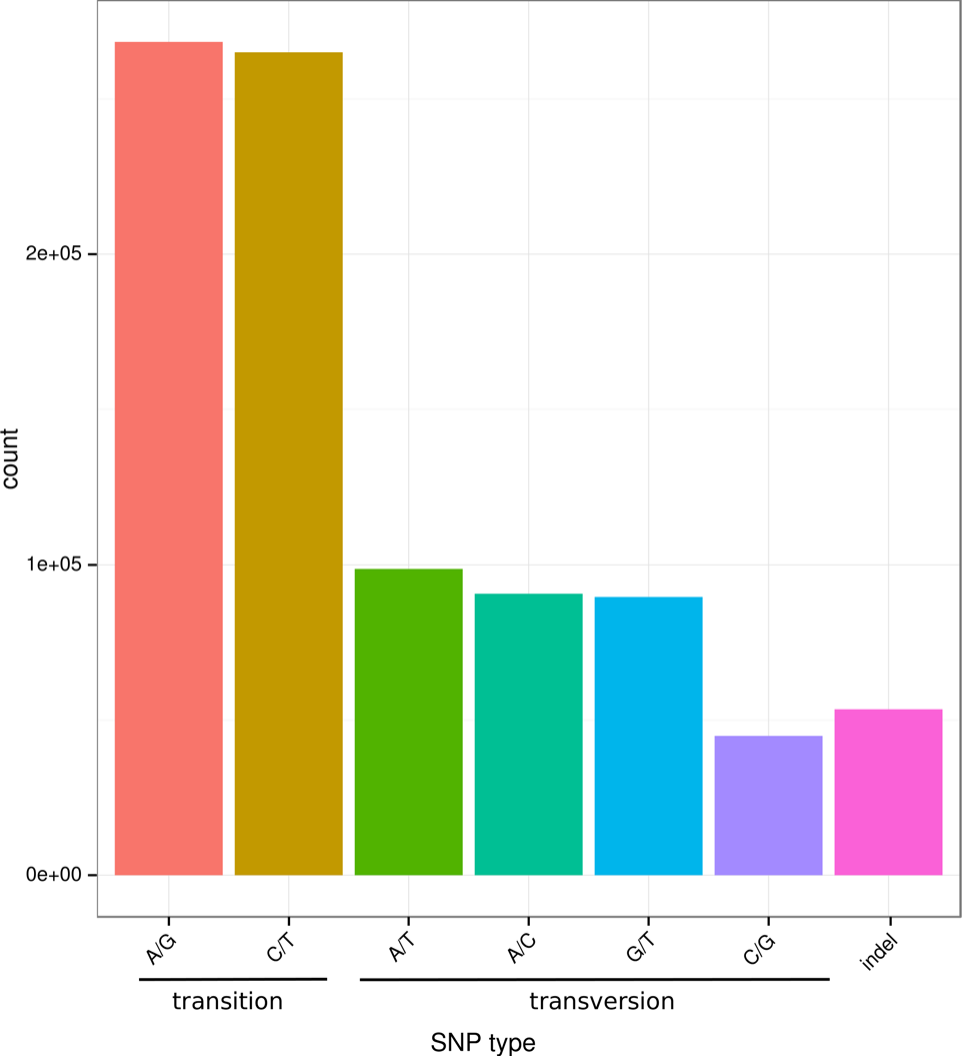
Frequency distribution of SNPs/InDels based on mutation types. The detected SNPs in transcriptome of *S*. *prenanti* were classified into transition and transversion, and the transition/transversion (Ts/Tv) ratio was estimated as ~1.65.

Small variants, including single nucleotide polymorphisms (SNPs) and insertion and deletions (InDels), are very useful markers for mapping important traits and for whole-genome association studies because of their wide distributions and abundant polymorphisms [15]. In the present study, we identified 857,535 SNPs (533,503 transitions and 324,032 transversions) and 53,481 InDels by mapping sequencing reads to 37,785 assembled unigenes. As is shown in Fig 7, the numbers of the two transition types A/G and C/T were similar, and the numbers of the transversion types A/T, A/C, and G/T were likewise similar; however, the transversion C/G and InDels represented the smallest types. This variation might be due to differences in base structure and hydrogen bond interactions between the base pairs [67]. The transition/transversion (Ts/Tv) ratio was about 1.65, which is comparable to the ratios reported in other fish species [11,68–70]. These SNP and InDel loci provide an abundant marker resource for investigating population genetic structures, wild population conservation, mapping important economic traits, and for performing association studies in *S*. *prenanti*.

## Conclusion

In this work, we sequenced mRNA fragments in spleen tissues and assembled the transcriptome into 48,517 transcripts and 37,785 unigenes. To our knowledge, this is the first transcriptome sequencing and *de novo* analysis of *S*. *prenanti* using the Illumina sequencing platform. By searching against known nucleotide and protein databases, 35,653 unigenes were successfully annotated. The 2,132 unigenes that failed to generate homologous hits may be non-coding RNAs, new genes, or species-specific sequences. Among the identified genes, more than 500 putative immune-related genes were identified in 16 signaling pathways. Most of the immune-related genes were reported for the first time and could provide important resources to understand the immune systems in *S*. *prenanti*. Additionally, 7,545 SSRs, 857,535 SNPs, and 53,481 InDels were identified from the transcriptome data. The transcriptome and molecular markers not only offer precise sequence information for the functional gene analysis, but also provide valuable marker resources for conservation and molecular assisted selection of *S*. *prenanti*.

## Supporting Information

S1 Fig Base quality distribution for the raw RNA-sequencing data. The six separate plots represent paired-end sequencing runs for the three RNA libraries.
(DOCX)

S2 Fig Gene annotation in Toll-like receptor signaling pathway using the KEGG database. Identified genes are highlighted by the green background.
(DOCX)

S3 Fig Gene annotation in complement and coagulation cascades using the KEGG database. Identified genes are highlighted by the green background.
(DOCX)

S4 Fig Gene annotation in the Chemokine signaling pathway using the KEGG database. Identified genes are highlighted by the green background.
(DOCX)

S1 Table Detailed annotation information for immune-related genes. (Included in a separate Excel file)
(XLS)

S2 Table SSR unit size distribution with different repeat numbers in the *S*. *prenanti* transcriptome. (Included in a separate Excel file)
(XLS)

S1 Text The list of 37,785 unigenes that were identified in the transcriptome of the S. prenanti. (FASTA)

S2 Text The gene lists of each category that have matches in NCBI Nr/Nt and Swiss-prot. (XLSX)
