# Supplementary material for: Identification of Immune-Related Genes and Development of SSR/SNP Markers from the Spleen Transcriptome of Schizothorax prenanti
Source: PLoS One. 2016 Mar 28;11(3):e0152572. doi: 10.1371/journal.pone.0152572 (PMC4809619; doi:10.1371/journal.pone.0152572)
Supplement: S1 Fig — The six separate plots represent paired-end sequencing runs for the three RNA libraries. (DOCX) [file pone.0152572.s001.docx]

**S1 Fig. Base quality distribution for the raw RNA-sequencing data.** The six separate plots represent paired-end sequencing runs for the three RNA libraries.

**
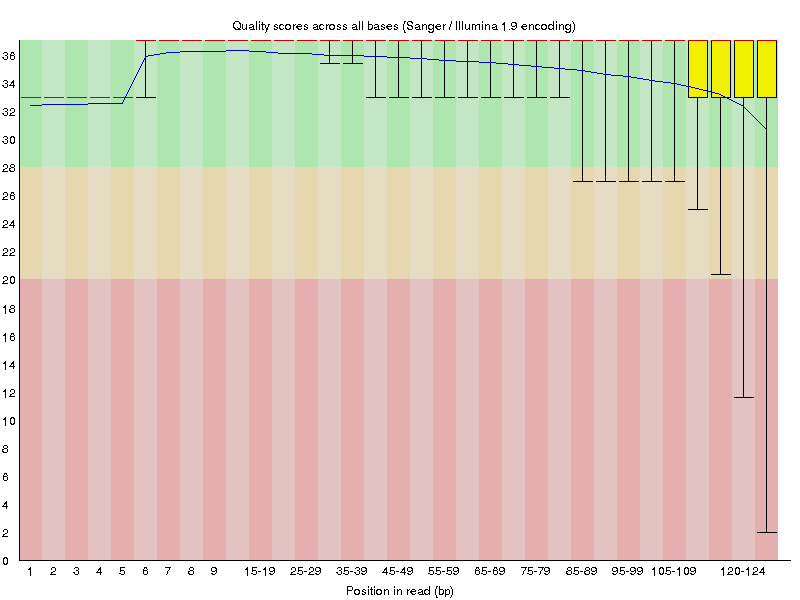

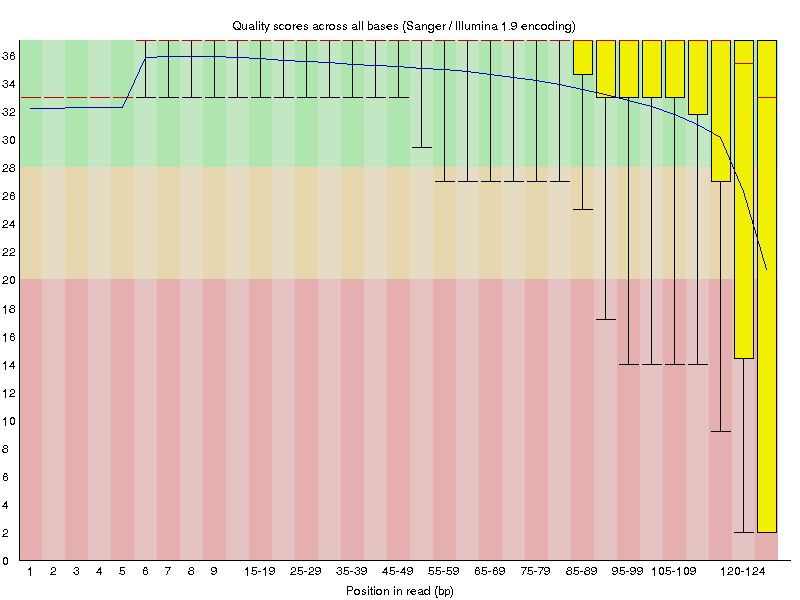
**

**
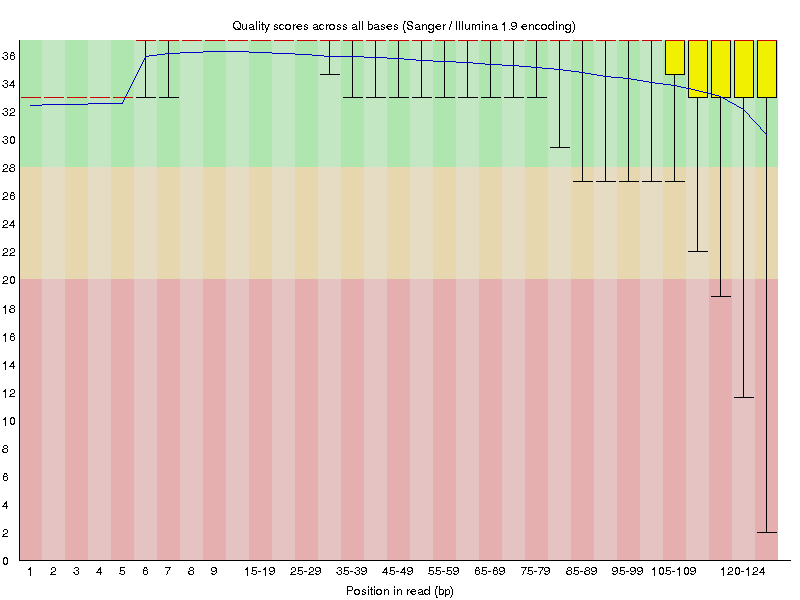

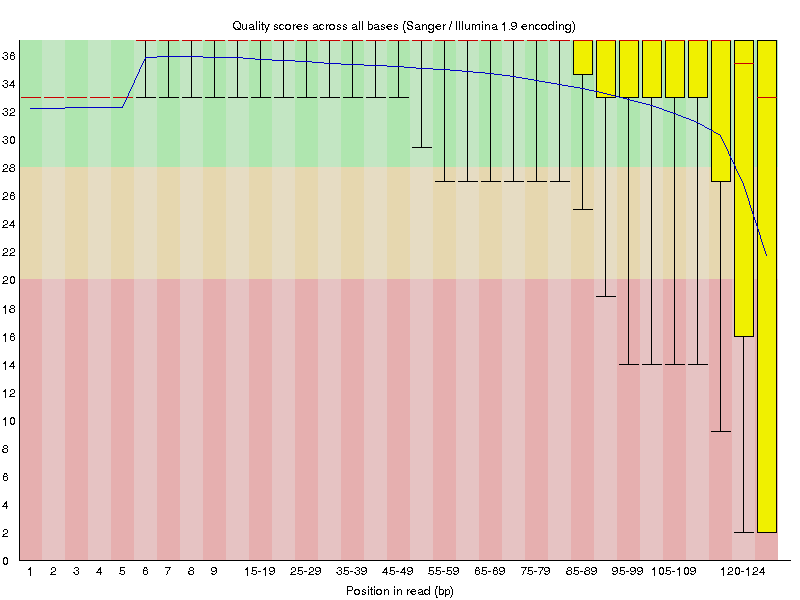
**

**
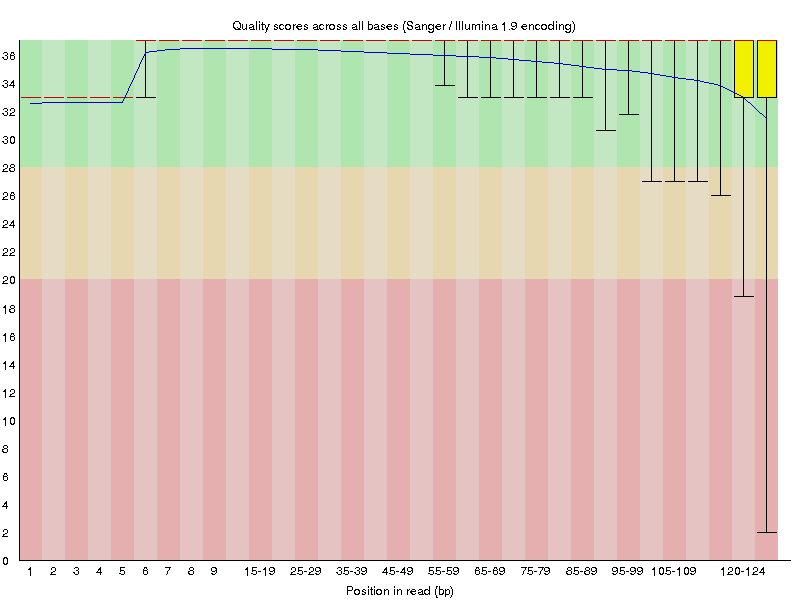

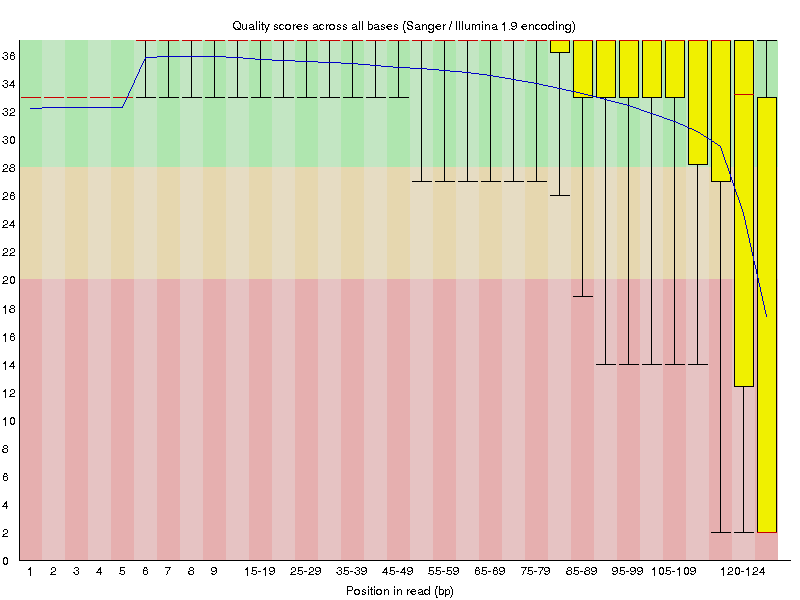
**
