# Supplementary material for: Identification of Immune-Related Genes and Development of SSR/SNP Markers from the Spleen Transcriptome of Schizothorax prenanti
Source: PLoS One. 2016 Mar 28;11(3):e0152572. doi: 10.1371/journal.pone.0152572 (PMC4809619; doi:10.1371/journal.pone.0152572)

**S3 Fig. Gene annotation in complement and coagulation cascades using the KEGG database.** Identified genes are highlighted by the green background.


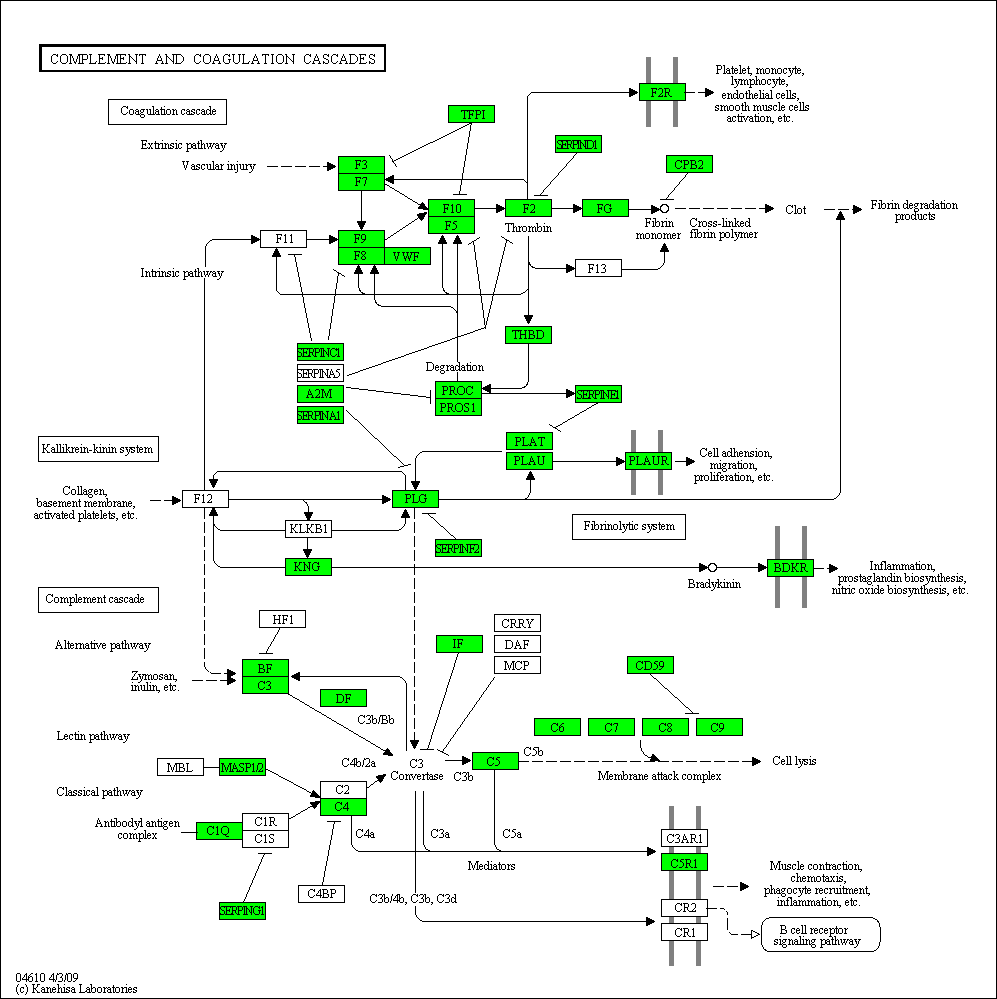

Supplement: S3 Fig — Identified genes are highlighted by the green background. (DOCX) [file pone.0152572.s003.docx]
